# GRP78/BIP/HSPA5 as a Therapeutic Target in Models of Parkinson's Disease: A Mini Review

**DOI:** 10.1155/2019/2706783

**Published:** 2019-03-05

**Authors:** Adaze Bijou Enogieru, Sylvester Ifeanyi Omoruyi, Donavon Charles Hiss, Okobi Eko Ekpo

**Affiliations:** ^1^Department of Medical Biosciences, University of the Western Cape, Robert Sobukwe Road, Private Bag X17, Bellville 7535, South Africa; ^2^Department of Anatomy, School of Basic Medical Sciences, College of Medical Sciences, University of Benin, Benin City, Edo State, Nigeria

## Abstract

Parkinson's disease (PD) is a common neurodegenerative disorder characterized by selective loss of dopamine neurons in the substantia nigra pars compacta of the midbrain. Reports from postmortem studies in the human PD brain, and experimental PD models reveal that endoplasmic reticulum (ER) stress is implicated in the pathogenesis of PD. In times of stress, the unfolded or misfolded proteins overload the folding capacity of the ER to induce a condition generally known as ER stress. During ER stress, cells activate the unfolded protein response (UPR) to handle increasing amounts of abnormal proteins, and recent evidence has demonstrated the activation of the ER chaperone GRP78/BiP (78 kDa glucose-regulated protein/binding immunoglobulin protein), which is important for proper folding of newly synthesized and partly folded proteins to maintain protein homeostasis. Although the activation of this protein is essential for the initiation of the UPR in PD, there are inconsistent reports on its expression in various PD models. Consequently, this review article aims to summarize current knowledge on neuroprotective agents targeting the expression of GRP78/BiP in the regulation of ER stress in experimental PD models.

## 1. Introduction

Parkinson's disease (PD) is a neurological disorder characterized by degeneration of dopaminergic neurons in the substantia nigra pars compacta (SNpc) of the midbrain, resulting in loss of dopamine in the striatum. In patients with PD, there are four primary motor symptoms which include tremor at rest, postural instability, rigidity, and bradykinesia [1]. PD was previously considered to be a condition that affects only the motor system, but with more research, it is now known to be a multifaceted disorder with diverse clinical features that include sleep, cognitive, and neuropsychiatric disorders [2, 3].

Although the etiology of the disease is not entirely understood, reports indicate that several factors such as oxidative and endoplasmic reticulum (ER) stress promote neuronal degeneration. The ER is regarded as the largest organelle in the cell with multiple functions such as protein, steroid, and phospholipid synthesis, storage of calcium, and metabolism of carbohydrates [4–8]. In the ER, chaperones such as 78 kDa glucose-regulated protein (GRP78), also known as binding immunoglobulin protein (BiP) or heat shock 70 kDa protein 5 (HSPA5) and other stress sensor proteins, are needed to maintain quality control of proteins. These proteins are activated to ensure proper handling and to prevent aggregation of misfolded/unfolded proteins [9]. Thus, when there is a disturbance in function, oxidative damage, or disruption of glucose or calcium homeostasis, the unfolded/misfolded proteins exceeds the folding capacity of the ER resulting to a condition commonly known as ER stress [10, 11]. The induction of ER stress and the consequent aggregation of misfolded or unfolded proteins have been implicated in PD pathogenesis [12, 13].

Existing treatment options for PD are inadequate as drugs are focused mainly on relieving symptoms. For example, levodopa is exceptionally effective for regulating PD symptoms, especially those linked to bradykinesia [14], and its combination with carbidopa improves the beneficial effects of levodopa. In cases where PD patients are sensitive to minor side effects such as nausea and vomiting, lodosyn may be taken with the routine carbidopa/levodopa therapy [15]. Other treatment options include dopamine agonists such as pramipexole [16], ropinirole [17, 18], and apomorphine [19, 20] while nondopaminergic drugs treatments include anticholinergics and amantadine [15] as well as entacapone [21] and tolcapone [22] catechol-o-methyl-transferase inhibitors.

Since there is no treatment for PD, there is an ever-increasing need to identify neuroprotective strategies with the ability to slow down or halt the advancement of PD. This search for new drug treatment options has paved the way for the discovery of such natural products as medicinal herbs, plant extracts, and their bioactive compounds. Some of these compounds are under clinical investigations owing to their remarkable potential as neuroprotective treatment options in PD [23, 24]. In this regard, while drug researchers are currently focused on discovering new remedies, plant-derived bioactive compounds targeting ER stress and its pathways could help in the identification and validation of novel treatment options in PD. Hence, this review presents an outline of the scientific literature on the research of plant-derived bioactive compounds and other neuroprotective agents targeting GRP78/BiP in experimental models of PD.

## 2. Endoplasmic Reticulum Stress Pathway and Disease

**Figure 1:**
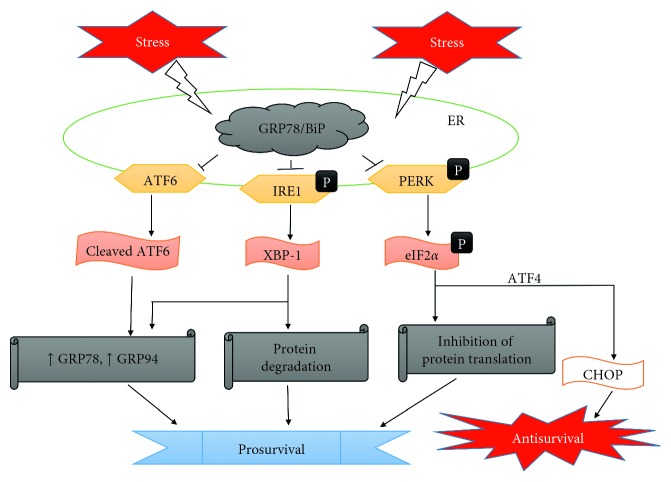
Simplified diagram highlighting the regulation of ER stress signaling pathways.

The ER stress pathway or unfolded protein response (UPR) is known to handle growing quantities of aberrant proteins in the ER [25]. This response program is tasked with the reduction of misfolded/abnormal proteins through various mechanisms (Figure 1). Firstly, GRP78/BiP disassociates from the ER stress sensors, namely, protein kinase RNA-like endoplasmic reticulum kinase (PERK), activating transcription factor 6 (ATF6), and inositol-requiring enzyme 1 (IRE1) to initiate the ER stress response. Following dissociation of GRP78/BiP, autophosphorylation and activation of PERK facilitate the phosphorylation of eukaryotic translation initiation factor 2a (eIF2a) to inhibit further protein synthesis and translation [26–28]. ATF6 is cleaved in the Golgi after translocation from the ER and then migrates into the nucleus to upregulate ER chaperones such as GRP78/BiP and 94 kDa glucose-regulated protein (GRP94) which enhances the folding capacity of the ER [29]. Also, IRE1 is involved in endoribonuclease activity and activates X-box binding protein 1 (XBP-1) to promote ER-associated degradation [30–32].

**Figure 2:**
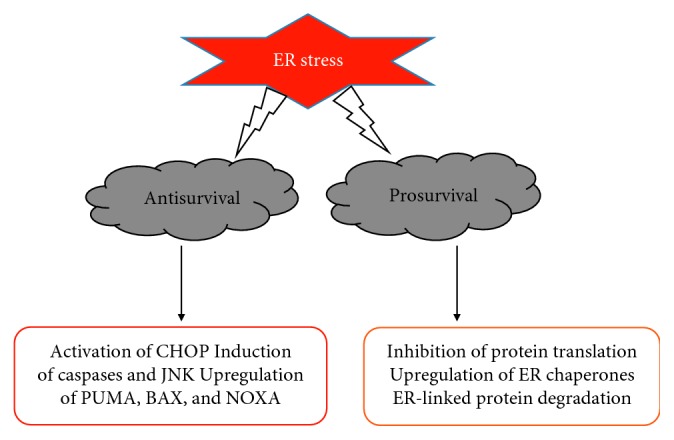
Important events during cellular response to ER stress.

The extent and degree of ER stress and UPR activation may determine if the ER stress response is either anti- or prosurvival (Figure 2). Certain aspects of the ER stress response such as increased expression of chaperones would appear to be advantageous by lessening the burden of misfolded proteins [33, 34], while other ER stress responses may be advantageous for a limited amount of time, thus leading to degeneration if sustained. Sustained activation of the UPR under stress would lead to apoptosis via the activation of ER-specific caspases, c-Jun amino-terminal kinase (JNK) and apoptosis signal-regulating kinase 1 (ASK1), induction of CCAAT-enhancer-binding protein homologous protein (CHOP), and the activation of p53 upregulated modulator of apoptosis (PUMA), BAX, and NOXA [35].

## 3. ER Stress Response in Parkinson's Disease

**Figure 3:**
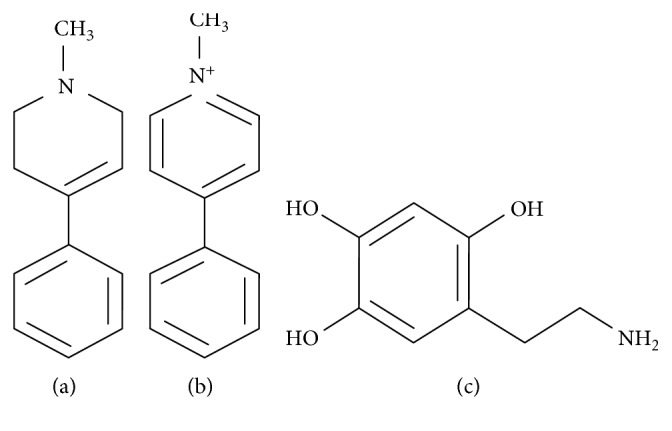
Diagram showing the chemical structure of PD toxins: (a) MPTP; (b) MPP^+^; (c) 6-OHDA.

GRP78/BiP is a key chaperone essential for proper functioning of the ER and in various cellular processes [36–38]. Most notable is its dual role of regulating protein folding and the initiation of UPR signaling in the ER [39]. In PD, there are inconsistent reports on the expression of GRP78/BiP in various experimental models. For instance, treatment of MN9D cells with a neurotoxin 1-methyl-4-phenylpyridinium (MPP^+^; Figure 3) resulted in a reduction of GRP78/BiP expression, while treatment of SH-SY5Y cells with a different neurotoxin 6-hydroxydopamine (6-OHDA; Figure 3) increased its expression [40, 41]. In a PD model using MPP^+^-treated rabbits, Ghribi and colleagues revealed the translocation of GRP78/BiP to the nucleus and cytosol from the ER as well as a significant decrease in TH-positive cells in the SNpc [42]. In a different study, Shimoke and coworkers demonstrated an increase in the expression of GRP78/BiP after exposure to tunicamycin; however, they observed no increase in the expression of GRP78/BiP in PC12 cells after treatment with a neurotoxin, 1-methyl-4-phenyl-1,2,3,6-tetrahydropyridine (MPTP; Figure 3), for 24 hours [43]. Duan and Mattson utilized the MPTP-treated mouse model of PD to demonstrate that the upregulation of GRP78/BiP by 2-deoxy-d-glucose significantly prevented loss of dopamine neurons [44].

In PD patients, GRP78/BiP was reported to be more expressed in the cingulate gyrus and parietal cortex when compared to healthy controls [45]. The upregulation of GRP78/BiP in the cingulate gyrus was linked to an increase in *α*-synuclein expression, thus providing an association between GRP78/BiP and *α*-synuclein toxicity. This observation is confirmed by a report demonstrating that the knockdown of GRP78/BiP aggravates the toxicity of *α*-synuclein in rats [46] and in another study showing that miRNA-induced reduction of GRP78/BiP enhanced cell death induced by a neurotoxin-rotenone [47]. In contrast to studies mentioned above, reports demonstrate that the upregulation of GRP78/BiP suppresses *α*-synuclein aggregation and toxicity in PD models [48, 49]. For example, Gorbatyuk and colleagues in a rat model of PD induced by an elevated level of human *α*-synuclein demonstrated that although the accumulation of *α*-synuclein induced the expression of apoptosis-regulating ATF4, the upregulation of GRP78/BiP inhibited *α*-synuclein toxicity by regulating ER stress signaling pathways [49].

Leucine-rich repeat kinase 2 (*LRRK2*) is the most significant gene mutated in PD [50]. *LRRK2* pathogenesis has been associated with ER stress as it partly localizes in the ER in dopaminergic neurons of individuals with PD [51]. Reports show that the neuroprotective activity of *LRRK2* against 6-OHDA or *α*-synuclein induced neurodegeneration in the nematode; *C. elegans* is attributed to the activity of GRP78/BiP via signaling through the p38 mitogen-activated protein kinase (MAPK) pathway [26, 52]. In confirmation of these reports, Samann and colleagues reported that *LRRK2* mutant *C. elegans* were highly vulnerable to ER stress and developed spontaneous neurodegeneration [53, 54]. Furthermore, ageing is the greatest risk factor for PD [55, 56], and various age-related changes in cellular structure and function are observed in PD patients. To corroborate these observations, studies reveal that ageing results in a significant reduction in the activity and expression of GRP78/BiP in the brain of old versus young rodents [57–59]. From the aforementioned, GRP78/BiP is undoubtedly an essential component of the UPR, and proper regulation of GRP78/BiP could prove valuable in identifying new treatment options in PD.

## 4. Regulation of GRP78/BiP by Therapeutic Agents in PD Models

Over the years, the use of neurotoxin-based experimental models of PD has contributed extensively to the understanding of PD and human health. For instance, such neurotoxins as MPTP, MPP^+^, 6-OHDA, paraquat, and rotenone have been utilized in the search, identification, and development of novel therapeutic agents in PD [60]. Also, the MPTP mouse and 6-OHDA rat models of PD have contributed immensely to the translation of animal experimentation into clinical practice and are still very much important for investigating different mechanisms of neuronal degeneration in PD. Considerable evidence shows that some experimental therapeutic agents have substantial antioxidant and anti-inflammatory activities, thus demonstrating an inhibitory effect in the oxidative and inflammatory mechanisms linked to neuronal loss in PD [61, 62].

The plant-derived bioactive compounds and other therapeutic agents highlighted in this review demonstrate significant neuroprotective effects and also regulate the activity of GRP78/BiP in experimental models of PD. One such compound is luteolin (3′, 4′, 5′, 7′-tetrahydroxyflavone), a naturally occurring flavonoid present in several herbs, fruits, and vegetables [63, 64]. It is a very potent antioxidant and is usually the most effective when compared to other flavonoids [65]. Plants containing luteolin have been utilized for the inhibition and treatment of such diseases as cancer and hypertension [66, 67]. Also, reports show that luteolin crosses the blood-brain barrier and has multiple biological, pharmacological, anticancer, anti-inflammatory, antibacterial, antiamnesic, and neuroprotective activities [68–71].

**Figure 4:**
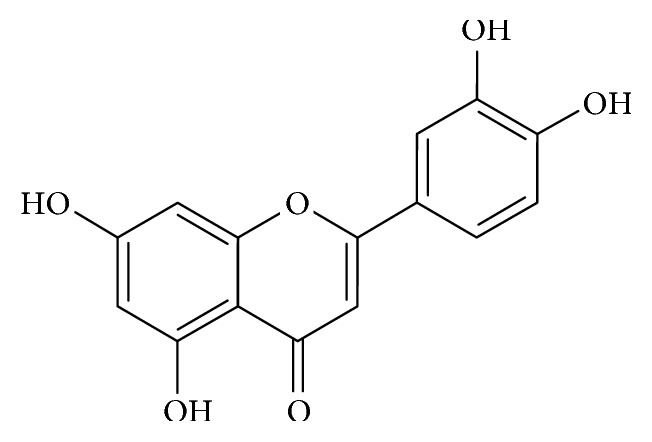
Diagram showing the chemical structure of luteolin.

While luteolin is structurally composed of hydroxyl groups at carbons 5, 7, 3′, and 4′ positions (Figure 4), the presence of 2-3 double bonds are linked to its multiple biological activities [72]. In a study, Hu and colleagues investigated the neuroprotective activity of luteolin in PC12 cells treated with 6-OHDA using RT-Q-PCR and western blot techniques [73]. They reported that luteolin attenuated the 6-OHDA-induced upregulation of GRP78/BiP and downregulated UPR, leading to the reduction of phospho-eIF2a, ATF4, and CHOP [73]. Based on these findings, the authors attributed the neuroprotective activity of luteolin to the regulation of GRP78/BiP and other UPR related proteins.

**Figure 5:**
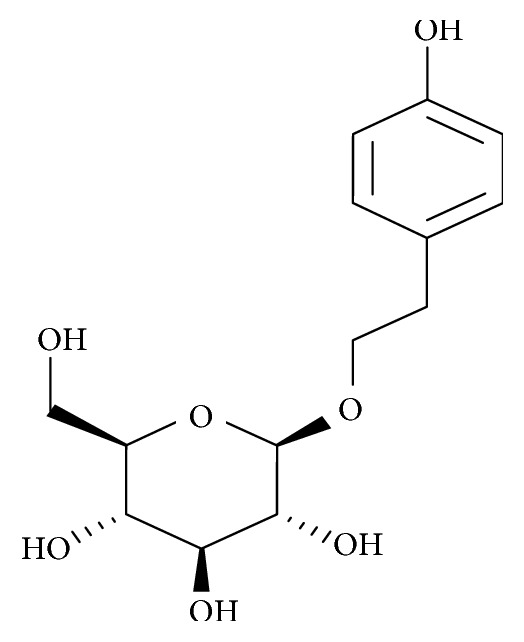
Diagram showing the chemical structure of salidroside.

Salidroside (*p*-hydroxyphenethyl-*β*-*D*-glucoside; C_14_H_20_O_7_; Figure 5), a phenol glycoside extracted as an active constituent from *Rhodiola rosea* L., is widely used in traditional folk medicine in Asia and Europe [74, 75]. In China, it is commonly used as an antifungal herb and as a supplement to improve kidney function, stimulate blood circulation, and clear chest congestion [76].

Salidroside exhibits a wide range of pharmacological activities including antioxidative, antiageing, anticancer, anti-inflammatory antitumour, antidepressive, antifatigue, adaptogenic, cardioprotective, and hepatoprotective effects [77–80]. In addition, reports show that salidroside is effective against cognitive decline during ageing and can protect neurons from apoptosis as well as mitochondrial dysfunction in experimental models of neurodegeneration [81–83]. To investigate salidroside's ability to regulate GRP78/BiP in an experimental model of PD, Tao and coworkers treated SN4741 cells with 6-OHDA after pretreatment with salidroside. Findings revealed that salidroside reduced the expression levels of GRP78/BiP and other ER stress markers (p-PERK and p-IRE1) when compared with cells treated with 6-OHDA only [84]. From the study, they demonstrated that the protective effect of salidroside against the toxicity of 6-OHDA was partly due to the regulation of GRP78/BiP and other ER stress markers.

**Figure 6:**
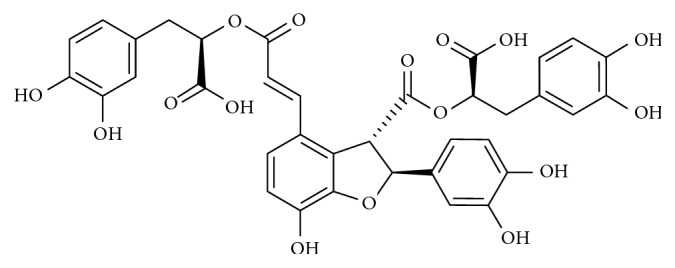
Diagram showing the chemical structure of salvianolic acid B.

Lithospermic acid (C_27_H_22_O_12_) is a key component of *Salvia miltiorrhiza*, a Chinese medicinal herb widely used to increase blood flow and treat diabetic as well as cardiovascular problems in humans [85]. Lithospermic acid shares a similar structure with salvianolic acid B (Figure 6) and is reported to have multiple pharmacological activities which include antihypertensive, antidiabetic, antiapoptotic, and antioxidant effects [86–88].

In a study by Lin and colleagues, MPP^+^-treated CATH.a cells were utilized as a model of PD to investigate the role of lithospermic acid on ER stress [89]. Findings from western blots revealed that MPP^+^ triggered ER stress in CATH.a cells by increasing the expression of GRP78/BiP, while lithospermic acid treatment inhibited the upregulation of GRP78/BiP, thus acting as a neuroprotective agent [89].

Basic fibroblast growth factor (bFGF), a member of the FGF family, is an essential protein with multiple physiological roles in the peripheral and central nervous system (CNS) [90, 91]. It is involved in a series of neurotrophic activities contributing to CNS repair and cell survival [92]. Reports indicate that bFGF shares receptors and influences a range of biological activities such as inhibition of apoptosis, cellular proliferation, and morphogenesis [93–95]. Previous studies show that bFGF exhibits neuroprotective activities in PD models; for instance, bFGF protected against rotenone-triggered dopaminergic cell loss in SH-SY5Y cells and enhanced survival of dopaminergic cells in human fetal tissue strands transplanted into immunosuppressed rats injected with 6-OHDA [96, 97]. In a study by Cai and coworkers, bFGF was found to suppress 6-OHDA-triggered upregulation of ER stress response proteins in Sprague–Dawley rats. Immunohistochemical and western blot findings revealed that bFGF treatment significantly inhibited 6-OHDA-induced increase in the expression of GRP78/BiP and CHOP, thus providing evidence on the regulation of GRP78/BiP as a neuroprotective mechanism in PD [98].

Ulinastatin (C_13_H_16_O_3_) is a glycoprotein and Kunitz-type serine protease inhibitor obtained by separation and purification from the urine of healthy men [99, 100]. Reports indicate that ulinastatin plays an important anti-inflammatory role through the inhibition of inflammatory cytokines and proteases [101]. For this reason, it is commonly used in Japan, Korea, and China for the management and treatment of severe pancreatitis, rheumatoid arthritis, and other inflammatory disorders [102–104]. Other pharmacological evidence reveals that ulinastatin has a protective role in multiple organ dysfunction syndrome, acute respiratory distress syndrome, and acute lung injury [105, 106]. To investigate the role of ulinastatin on ER stress in an *in vivo* model of PD, Li and colleagues observed that paraquat triggered a higher expression of GRP78/BiP and apoptosis in treated rats when compared to rats in the control group. However, they observed that ulinastatin-treated rats showed lower expression of GRP78/BiP when compared to rats treated with paraquat only. These findings demonstrated that the regulation of GRP78/BiP by ulinastatin was partly responsible for its overall protective effect observed in the PD model [107].

**Figure 7:**
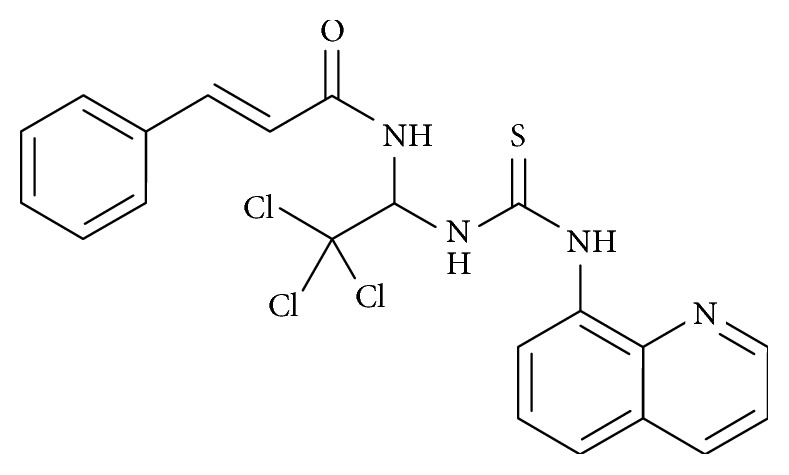
Diagram showing the chemical structure of salubrinal.

Salubrinal (C_21_H_17_Cl_3_N_4_OS; Figure 7) is a synthetic compound that was discovered in a screening of compounds with protective activity on ER stress-mediated cell death [108]. It is used experimentally to investigate stress response studies linked to eIF2*α* [109], and as a mechanism of action in ER stress, it inhibits the dephosphorylation of eIF2*α*, leading to a reduction in protein synthesis and inhibition of protein translation [110, 111]. Reports indicate that salubrinal is protective against cell death induced by tunicamycin, arsenic, cyclosporine, cadmium, hypoxia, and paraquat [112–115].

As a neuroprotective agent in PD, salubrinal prevented MN9D cells from MPP^+^ and 6-OHDA-induced toxicity [116]. It is believed that the protective activity of salubrinal can be attributed to the functional groups in its molecular structure except for the quinolone moiety [117]. To demonstrate the neuroprotective mechanism of salubrinal on paraquat-induced ER stress in SH-SY5Y cells, Yang and colleagues showed that salubrinal inhibited the activation of GRP78/BiP and other ER stress sensors IRE1, ASK1, JNK, and CHOP [118]. In a different study by Goswami and colleagues, treatment of neuro-2A cells with rotenone increased the expression of GRP78/BiP and CHOP [119]; however, pretreatment of the cells with salubrinal reduced the expression of GRP78/BiP and CHOP. The authors highlighted the inhibition of eIF2*α* dephosphorylation and the regulation of GRP78/BiP as a neuroprotective mechanism of salubrinal in rotenone-induced toxicity in PD [119].

**Figure 8:**
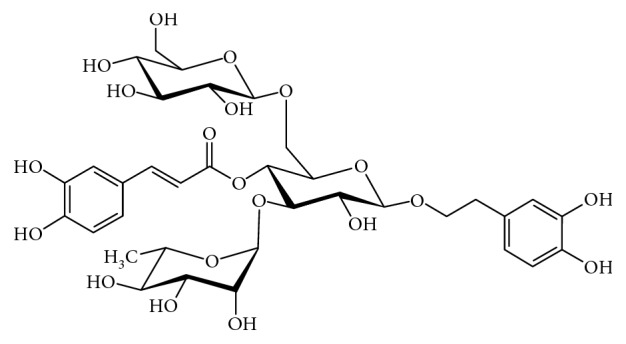
Diagram showing the chemical structure of echinacoside.

Echinacoside (C_35_H_46_O_20_; Figure 8) is a primary component of phenylethanoid glycoside isolated from *Cistanche salsa*, a Chinese herbal medicine known for its antifatigue and antisenile properties [120, 121]. Reports show that echinacoside has potent antioxidant effects, scavenges for free radicals, and inhibits macrophage-induced generation of nitric oxide [122–124]. Other biological effects include anti-inflammatory, antiageing, antitumour, and hepatoprotective activities [125, 126].

The role of echinacoside in neurodegenerative disorders has also been reported; for instance, studies show that echinacoside treatment significantly protected PC12 and SH-SY5Y cells against H_2_O_2_ and TNF-*α* triggered cell death, respectively [127, 128]. In another study, echinacoside prevented dopaminergic neuronal loss in rats injected with 6-OHDA and mitigated the reduction of dopamine and its intermediates [129, 130]. Geng and colleagues demonstrated that echinacoside improved behavioural deficits, prevented loss of dopamine neurons, and reduced the activation of caspase 3/8 in *in vivo* and *in vitro* models of PD [131]. Also, Wang and coworkers reported that echinacoside prevented 6-OHDA-triggered loss of dopamine neurons via the attenuation of ROS generation and mitochondrial dysfunction [121]. To elucidate the role of echinacoside in the regulation of GRP78/BiP in an experimental model of PD, Zhang and colleagues revealed that echinacoside attenuated the upregulation of GRP78/BiP in 6-OHDA-treated PC12 cells and Sprague–Dawley rats injected with 6-OHDA, thus highlighting the neurotherapeutic potential of echinacoside in experimental models of PD [132].

**Figure 9:**
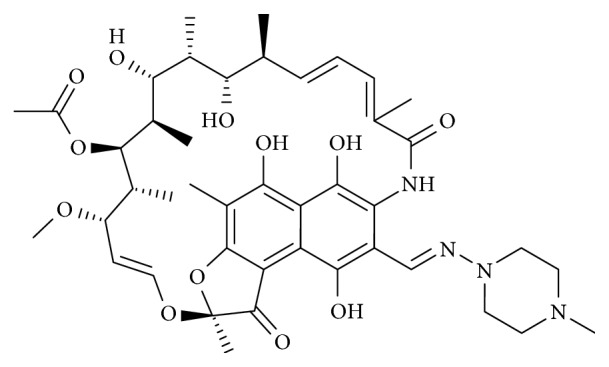
Diagram showing the chemical structure of rifampicin.

Rifampicin is derived from rifamycins, a class of antibiotics obtained from *Nocardia mediterranei* through a process of fermentation [133]. It is commonly used against *Mycobacterium tuberculosis* and other mycobacterial infections [133, 134]. Its hydroxyl radical scavenging properties are ascribed to the naphthohydroquinone ring in its chemical structure (Figure 9), while its lipophilic ansa chain is believed to help in its transport into the brain parenchyma across the blood-brain barrier [135, 136]. Pharmacological reports show that rifampicin has immunosuppressive and antioxidant properties [137–139] and inhibits *β*-amyloid accumulation and neurotoxicity [140]. It also prevents lipopolysaccharide-triggered upregulation of proinflammatory mediators, decreases NF-*κ*B and MAPK signaling [134, 141], attenuates apoptosis in focal ischemic stroke, and inhibits loss of dopaminergic neurons in PD models [133, 142, 143].

To investigate the primary mechanism by which rifampicin promotes neuronal survival, Jing and colleagues revealed a dose-dependent activation of GRP78/BiP in rifampicin-treated PC12 cells [144]. Upon silencing of the GRP78/BiP gene, they investigated if rifampicin-induced GRP78/BiP activation protected against toxicity in rotenone-treated PC12 cells. Western blots and morphological evaluation revealed that cells without the GRP78/BiP gene were more prone to rotenone-triggered damage when compared to cells with the GRP78/BiP gene irrespective of rifampicin treatment [144]. These findings show that silencing of the GRP78/BiP gene mitigated rifampicin-induced protection and thus confirmed that the regulation and activation of GRP78/BiP was responsible for the neuroprotective activity of rifampicin in the PD model.

## 5. Conclusion

Protein misfolding and aggregation is implicated in the pathogenesis of PD, and the regulation of GRP78/BiP is critical for proper functioning of the UPR. As highlighted in this review, several studies have attempted to unravel the mechanism behind ER stress by targeting GRP78/BiP and the UPR as a way of halting dopaminergic neuronal loss in PD. Although it is established that GRP78/BiP is an essential chaperone in the UPR, studies discussed in this review indicate that the expression of GRP78/BiP is altered in various models of PD depending on the cell type and toxin used in inducing neuronal damage. Consequently, various neuroprotective agents induce the upregulation or downregulation of GRP78/BiP in response to the ER stress-inducing agent in these PD models to promote the survival of dopaminergic neurons. Also, evidence from this review indicate that a translational potential exists for the regulation of GRP78/BiP activity; however, further investigations are needed to properly understand the involvement of GRP78/BiP in the protection of neurons against degeneration in PD. This knowledge would be valuable in designing novel remedies targeted at combating PD and other neurodegenerative disorders linked to the aggregation of misfolded proteins.
